# Advancements in Nanomaterial Dispersion and Stability and Thermophysical Properties of Nano-Enhanced Phase Change Materials for Biomedical Applications

**DOI:** 10.3390/nano14131126

**Published:** 2024-06-29

**Authors:** Qian Zhang, Tkhu Chang Le, Shuang Zhao, Chenxi Shang, Menglin Hu, Su Zhang, Yushi Liu, Shuang Pan

**Affiliations:** 1 The First Affiliated Hospital of Harbin Medical University, No. 23 Youzheng Street, Nangang District, Harbin 150001, China; qianzhang@hrbmu.edu.cn (Q.Z.); trang020595@gmail.com (T.C.L.); 2023021157@hrbmu.edu.cn (S.Z.); 2023021164@hrbmu.edu.cn (C.S.); 2022021059@hrbmu.edu.cn (M.H.); 2022021057@hrbmu.edu.cn (S.Z.); 2 School of Stomatology, Harbin Medical University, No. 143 Yiman Street, Nangang District, Harbin 150001, China; 3 School of Civil Engineering, Harbin Institute of Technology, Harbin 150090, China

**Keywords:** phase change material, nanomaterials, thermophysical properties, biomedical application

## Abstract

Phase change materials (PCMs) are materials that exhibit thermal response characteristics, allowing them to be utilized in the biological field for precise and controllable temperature regulation. Due to considerations of biosafety and the spatial limitations within human tissue, the amount of PCMs used in medical applications is relatively small. Therefore, researchers often augment PCMs with various materials to enhance their performance and increase their practical value. The dispersion of nanoparticles to modify the thermophysical properties of PCMs has emerged as a mature concept. This paper aims to elucidate the role of nanomaterials in addressing deficiencies and enhancing the performance of PCMs. Specifically, it discusses the dispersion methods and stabilization mechanisms of nanoparticles within PCMs, as well as their effects on thermophysical properties such as thermal conductivity, latent heat, and specific heat capacity. Furthermore, it explores how various nano-additives contribute to improved thermal conductivity and the mechanisms underlying enhanced latent heat and specific heat. Additionally, the potential applications of PCMs in biomedical fields are proposed. Finally, this paper provides a comprehensive analysis and offers suggestions for future research to maximize the utilization of nanomaterials in enhancing the thermophysical properties of PCMs for biomedical applications.

## 1. Introduction

**Figure 1:**
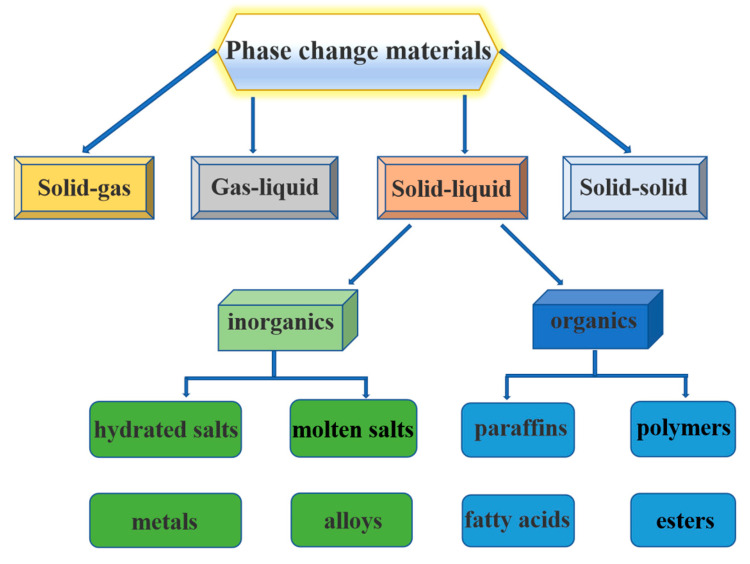
Classification of phase change materials (PCMs).

Phase change material (PCM) is a kind of material that incurs phase transition at a specific temperature accompanied by a large amount of heat absorption or release [1]. The most common classification of solid–liquid PCMs can be divided into inorganic and organic PCMs based on the different chemical compositions. Under these two categories, solid–liquid PCMs are further classified, as shown in Figure 1. PCM has the two characteristics of constant temperature and high latent heat value when phase transition occurs, which means that it can be used in the biological field to achieve accurate and controllable temperature regulation. There are lots of therapeutic methods related to temperature control in the biomedical domain [2]. For example, mild heat (±42 °C) can used for tissue regeneration [3]; tumor cells are more susceptible to moderate heat (±45 °C) compared to normal cells [4,5,6]; and hyperthermia (>50 °C) is more effective in treating infections [7]. Therefore, photo-thermal effects can be manipulated for various therapies according to different temperatures. PCM can be utilized to precisely operate the local temperature distribution of a lesion within a specific narrow range to avoid thermal injury of the adjacent normal tissues. Wang et al. [8] introduced eicosane as a PCM and micro-encapsulated it in an acrylic functionalized silica shell through lotion-templated interface polymerization. This design was used to control the release of drugs, and it could also provide thermal cushioning for thermosensitive chemicals or biomolecules when exposed to violent, highly fluctuating ambient temperatures. Chen et al. [9] designed a functional mask composed of a flexible layered frame-based polyethylene glycol (PEG) PCM composite for heat energy collection and hyperthermia. This respirator could hold the air flow entering the cavum nasi at 40–43.5 °C for 30 min and effectively alleviate rhinitis. However, it was found that the amount of PCMs used in medical applications is relatively small because of the biosafety and spatial limitations within human lesion tissues, but, in order to ensure the heat transfer and storage effect, there is an urgent need to improve the thermophysical properties of PCMs.

However, most PCMs have their own limitations. For common organic PCMs, the thermal conductivity is often relatively low, and lower thermal conductivity also means that the heat transfer efficiency is poor [10]. For another common PCM, inorganic crystalline hydrates, although the heat transfer performance is much better than that of organic PCMs, they still face the problem of extensive supercooling caused by incongruous melting [11]. In addition, compared with other thermal energy storage forms, the most outstanding performance advantage of PCMs is the latent heat storage during phase change, which is also the main thermal energy storage method of PCMs [12]. A higher latent heat value of PCMs means a higher thermal storage capacity, demonstrating greater application value [13,14].

In response to these circumstances, a range of methods have been employed to improve the properties of phase change materials (PCMs). For instance, the incorporation of nanomaterials can augment thermal conductivity, thereby imparting additional functionalities to PCMs such as photothermal and electrothermal conservation [10]. Therefore, in order to obtain PCMs with good thermal storage performance, researchers have been committed to studying their influence on the phase change latent heat of PCMs. For PCMs, the display of heat storage capacity not only includes latent heat but also specific heat (sensible heat), which is another important form of heat storage. Unlike latent heat storage, which concentrates on a specific temperature point to store and release large amounts of heat energy, specific heat storage refers to storing and releasing smaller amounts of thermal energy in addition to the phase change process [15]. However, considering that the actual application temperature range of PCMs is usually relatively wide, aside from the phase change temperature point, the form of heat storage provided by PCMs is specific heat storage [16]. Therefore, in order to obtain PCMs with more excellent and comprehensive energy storage capacity, improvement of the specific heat also plays an important role [17]. At present, the improvement of the thermophysical performance of PCMs mainly focuses on the following four points: the enhancement of thermal conductivity, latent heat, specific heat, and the reduction of supercooling.

Recently, research on nanomaterials has made more extensive breakthroughs [18]. Nanostructured materials can be classified as unidimensional, two-dimensional, and three-dimensional nanomaterials, according to their dimensions (most nanoparticles are three-dimensional materials). According to the chemical category, they can also be divided into metal nanomaterials, metal oxide nanomaterials, inorganic non-metallic nanomaterials, and carbon nanomaterials [19].

With the maturity of nanomaterial preparation processes, there are more reports on PCMs modified by nanoparticles to obtain better thermophysical properties [20]. Nanoparticles with high thermal conductivity can better enhance the thermal conductivity of PCMs with a small amount of doping, thus reducing the heat storage and release time of PCMs and increasing the working efficiency of the heat storage system [21]. Moreover, nanoparticles have a large specific surface area. Some nanoparticles possess multiple functional groups on their surfaces, making them easy to interact with PCMs, thereby altering the latent heat and specific heat performance of PCMs [22]. In conclusion, to comprehensively introduce the role of nanomaterials in addressing the limitations and enhancing the performance of PCMs, this paper will respectively discuss the dispersion and stability of nanoparticles in PCMs (Section 2), as well as their effects on thermophysical properties including thermal conductivity, specific heat capacity, and latent heat (Section 3). Furthermore, based on detailed investigations, this paper will thoroughly present applications in the medical field such as drug delivery, medical dressing, and healthcare, etc. (Section 4).

## 2. Dispersion and Stability of Nanomaterials in PCMs

### 2.1. Agglomeration of Nanomaterials and Their Influence Factors

Many studies have shown that nanomaterials are easy to agglomerate in various dispersion media [23]. If the dispersion and stability of nanoparticles in PCMs are poor, the effectiveness of the nanomaterials’ modification may decline over the long-term thermal cycling of PCMs. “Stability” in the context of dispersed composites involving nanomaterials refers to the collective ability of a composite to maintain a uniform dispersion, resist chemical and physical degradation, and retain performance over time. Methods to evaluate the dispersion stability of nanoparticles in PCMs mainly include sedimentation, particle size detection, zeta potential measurement, transmittance, and electron microscope observation, etc. [24].

The dispersion and stability of nanoparticles in PCMs involve several key aspects. First, the unique properties of nanoparticles are largely influenced by their size. Nanoparticles have a large specific surface area due to the arrangement of atoms on their surface, making them more prone to adsorb other substances. Secondly, nanoparticles contain numerous crystal defects such as twins and dislocations, leading to a high number of unsaturated bonds. This increases the number of active sites on the particle surface, resulting in high chemical activity. Due to their multiple activation sites, nanoparticles easily combine with other atoms to achieve a more stable state. Thirdly, the wettability between nanoparticles and the PCM dispersion medium is crucial and is primarily influenced by the hydrophilic and hydrophobic properties of the nanoparticles [25,26,27].

### 2.2. Improvement Methods

The methods to enhance the dispersion and stability of nanoparticles in PCMs can be divided into the following three categories according to their principles: enhancing the wettability of nanoparticles on the surface of PCMs, enhancing the electrostatic repulsion between nanoparticles, and using polymer materials to adsorb nanoparticles to form a three-dimensional protective layer [23]. According to the implementation methods, they can be divided into physical and chemical methods. Currently, the following methods are commonly used to enhance the dispersion and stability of nanoparticles in PCM.

#### 2.2.1. Physical Methods

(1) Mechanical dispersion method

The mechanical dispersion method involves directly dispersing nanoparticles into the dispersion medium through high-speed stirring, ball milling, grinding, etc. During the mechanical dispersion process, the dispersant forms a molecular film on the surface of the particles, which prevents them from coming into contact with each other. This creates a spatial barrier that effectively prevents the aggregation of nanoparticles.

The dispersant is also used to form a layer of molecular film on the particle surface, which hinders the mutual contact between particles, increases the distance between particles, and plays a certain role in steric hindrance, thus effectively preventing the agglomeration of nanoparticles [1]. The advantage of the mechanical force method is that the output of nanoparticles is high and the process is simple and easy to control [28]. The vast majority of dispersion means involve the mechanical dispersion method, which can also be combined with other enhanced dispersion means.

The dispersion effect of the mechanical dispersion method heavily relies on the characteristics of the nanoparticles themselves. Typically, it is necessary to apply dispersants in conjunction to achieve a better dispersion effect [23,29]. However, the potential impact of dispersants on the properties of PCMs should be taken into consideration when adding them to the PCM. Generally, the introduction of dispersants tends to reduce the heat storage capacity of PCMs. Therefore, when incorporating dispersants, it is important to evaluate their potential effects on PCM performance.

(2) Ultrasonic dispersion

Ultrasound possesses unique dispersion properties that induce high pressure and stress within a liquid medium. When exposed to ultrasonic energy, shear processes disrupt the binding energy of nanoparticles [30]. Sonification also triggers two nonlinear effects—cavitation and sound streaming—which impede nanoparticle agglomeration in the liquid, thereby facilitating their separation. [31].

Ultrasonic dispersion finds extensive application in the preparation of nano/micron lotions and the dispersion of nanoparticles [32,33,34]. It involves delivering energy to the nanoparticle PCM system. However, when ultrasonic power and duration exceed optimal levels, excessive thermal and mechanical energy can result, leading to increased collisions between nanoparticles and subsequent agglomeration. Hence, it is crucial to avoid excessive heat during the ultrasonic dispersion process.

(3) Dispersants

A dispersant is a commonly used auxiliary material for dispersing nanomaterials. There are two main working principles of dispersants. One is electrostatic stability, that is, after polar molecules are adsorbed on the surface of nanomaterials, nanomaterials carry the same charge, thus repelling each other and achieving electrostatic balance [35]. The other principle is the steric hindrance stabilization mechanism, which is mainly applicable to many non-polar polymers. The stabilization mechanism means that the hydrophobic (hydrophobic) group of the polymer is adsorbed on the surface of the nanoparticles and the hydrophilic (hydrophobic) group is suspended in polar (nonpolar) solvents to maintain a good dispersibility of nanoparticles in the matrix phase. The approaching of two nanoparticles compresses the polymer adsorption layer and decreases the number of polymer molecular chain configurations and the configuration entropy. This leads to the increased free energy of the system, thus resulting in repulsive potential energy. Moreover, the concentration of polymer within the overlapping area is increased due to the interpenetration and overlapping of adsorption layers, and the repulsive potential energy is generated by the formation of osmotic pressure, thus dispersing the nanomaterials [36].

#### 2.2.2. Chemical Methods

Chemical methods to enhance the dispersion effect of nanomaterials typically involve two approaches: first, the preparation of nanomaterials in the dispersion medium, and second, the modification of the surface of nanomaterials. The former method is often limited by the characteristics of the dispersion medium and is applicable in relatively few scenarios. The latter, however, is currently the most significant means of modifying nanomaterials [37].

The surface treatment of nanoparticles involves introducing functional groups onto the surface of nanomaterials through chemical processes. These functional groups are often compatible with the dispersion medium, facilitating the improved dispersion of nanoparticles within it [38,39].

(1) Coupling agent method

The coupling agent method stands as the most widely employed approach for surface treatment. This method operates on the principle that ionic groups and small molecular substances, formed after the hydrolysis of coupling agent molecules, interact with the hydrogen bonds and hydroxyl groups present on the surface of nanoparticles. This interaction leads to alterations in the surface properties of the nanoparticles [40].

**Figure 2:**
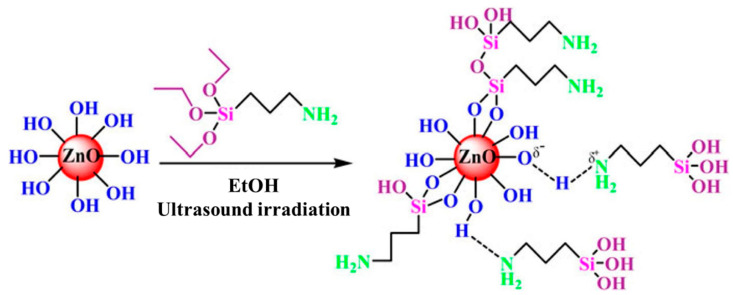
Principle for grafting silane coupling agent APTES onto ZnO nanoparticles [42]. Reprinted/adapted with permission from Ref. [42]. Copyright 2011 Springer Nature.

Let us take silane coupling agent (SCR) as an example. Silane coupling agent is the most commonly used modifier to modify the surface of nano-metal oxides and is an organosilicon compound with the general formula of (X-(CH_2_)_n_-Si-R_3_) [41]. Figure 2 shows the principle of grafting the silane coupling agent APTES onto ZnO nanoparticles. The nanoparticles’ surface naturally contains hydroxyl (-OH) groups due to oxidation, facilitating the formation of hydrogen bonds with the silane group of the coupling agent. Alternatively, the binding process can involve initially attaching the X group in the SCA (e.g., the amine group in APTES) to the nanoparticle surface via hydrogen bonding. Typically, the coupling agent on the surface can be fixed through aging or heat treatment. The resulting interface comprises a blend of hydrogen bonds, ionic bonds, and covalent siloxane bonds [42].

(2) Esterification modification

The esterification reaction involves the reaction of metal oxide with alcohol. This reaction is utilized to modify the surface of nanoparticles, converting the original hydrophilic or hydrophobic surface to one that is hydrophilic or hydrophobic. This surface functionalization is crucial for practical applications. The esterification reaction is particularly effective in modifying the surfaces of nanoparticles with weakly acidic or neutral characteristics, such as SiO_2_. Additionally, carbon nanoparticles can also undergo surface modification through esterification [43].

(3) Polymer grafting method

An alternative approach to functionalize nanoparticles involves grafting polymers onto their surface. Compared to other treatment methods, polymer grafting offers the advantage of achieving a more uniform surface modification. Furthermore, this method allows for the alteration or introduction of various surface properties by selecting suitable monomers to graft different chemical functional groups onto the particles.

The grafted polymers, sometimes referred to as “polymer dispersants”, vary in molecular weight. To prevent particle aggregation, the length of the polymer chain must be optimized. A short polymer chain may not provide a sufficiently thick barrier to overcome attractive van der Waals forces, while a long polymer chain can lead to particle bridging and flocculation [44].

## 3. The Effect of Nano-Modification on the Thermophysical Properties of PCMs

### 3.1. Thermal Conductivity

Nanomaterials exhibit excellent thermal conductivity, leading many scholars to believe that combining them with materials of poor thermal conductivity can enhance overall thermal conductivity [45,46]. Additionally, nanoparticles possess a large specific surface area, effectively increasing the heat exchange area compared to conventional modification methods such as fins [47].

The addition of nanomaterials generally enhances the thermal conductivity of phase change materials (PCMs). However, the factors influencing the thermal conductivity of PCMs modified by different types of nanoparticles vary. The following section will discuss the impact of various nanoparticles on the thermal conductivity of PCMs.

#### 3.1.1. Nano Metal

It is well known that metals are excellent conductors of heat. Among metals, silver has excellent thermal conductivity; its thermal conductivity is about 430 W/(m·K), which is close to that of gold and copper. However, silver and gold are very expensive and also highly active and easy to oxidize. Copper is low-cost and offers advantages over silver and gold in many applications. In recent years, these metals have been processed into nanomaterials to modify PCMs, yielding good results.

**Figure 3:**
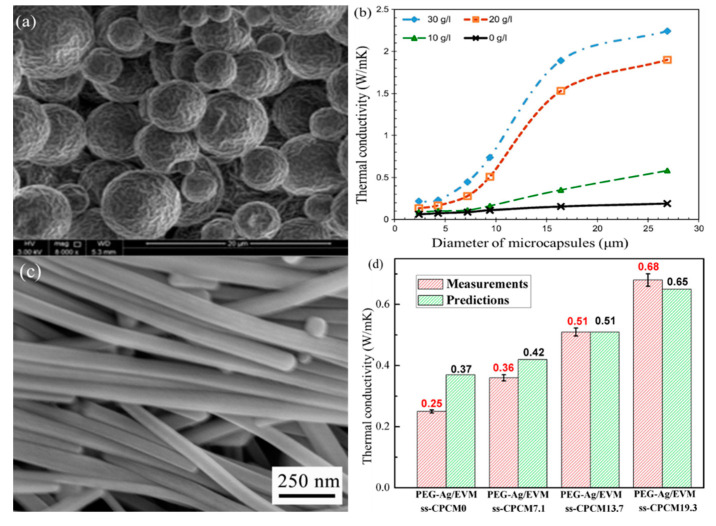
(**a**) SEM image of nano-Ag shell (The scale represents 20 μm), (**b**) thermal conductivities of nanocapsules with different diameters [48], (**c**) SEM image of the AgNWs, and (**d**) the measured and predicted thermal conductivities of the composite form-stable PCM [49]. Reprinted/adapted with permission from Ref. [48]. Copyright 2016 Elsevier. Reprinted/adapted with permission from Ref. [49]. Copyright 2016 Elsevier.

Al-Shannaq [48] used a nano-thick Ag shell to increase the thermal conductivity “*k*” of PCM by 1168%. The microstructure of the nano-Ag shell and the thermal conductivities of the microencapsulated PCM are shown in Figure 3a,b, respectively. The thermal conductivity of the shell of the microcapsule PCM was poor, hindering its performance in heat transfer and energy storage. To improve the thermal conductivity (kk) of the microencapsulated PCM, a metal shell was used to cover the microcapsules by activating the surface of multiple metals and applying electroless plating. The effectiveness of this modification method depended on the area of the metal shell and whether the nano-scale metal shell outside the microcapsule could form a thermal conduction path. The heat conduction performance was greatly improved once the thermal conduction path was established.

Deng et al. [49] prepared nano-silver wires (AgNWs) and introduced them into the expanded graphite–PCM composite, using the nano-silver wires to create a heat conduction path. Figure 3c shows an SEM image of the AgNWs. The study found that using silver nanowires with lengths of 5 to 20 μm and diameters of 50 to 100 nm could greatly improve the thermal conductivity of composite PCMs, as shown in Figure 3d. This approach increased the thermal conductivity by 10.3 times. Zeng et al. [50] developed silver nanowires to prepare silver-doped PCM nanocomposites. Using AgNWs, the thermal conductivity was increased by 356% to 380% with the addition of 45 wt% AgNWs. Additionally, the thermal conductivity could be enhanced by two to three times in graphene-doped PCM.

Although silver nanomaterials have excellent properties and achieve great modification effects, their high price makes them difficult to popularize. Copper nanomaterials, on the other hand, are relatively low-cost. Various nanowires and nanoparticles made of copper are mixed into the preparation of composite PCMs, significantly improving their thermal conductivity. Shah et al. [51] used Cu nanowires to improve the thermal conductivity of PCM. By adding 0.17 wt% copper nanowires, the thermal conductivity of calcium chloride hexahydrate could be increased by 160%. Molefi et al. [52] used copper nanoparticles to increase the thermal conductivity of paraffin PCM by 70%. As the content of the copper nanoparticles increased, the thermal conductivity of the paraffin increased almost linearly. Tang et al. [53] studied the increase in the thermal conductivity of a composite form-stable PCM doped with copper nanoparticles, with PEG embedded in SiO_2_. Compared to pure PCM, a 38.1% increase in thermal conductivity was achieved by adding only 2.1 wt% copper nanoparticles.

#### 3.1.2. Nano-Metal Oxide

Metal oxides, such as aluminum oxide and copper oxide, exhibit good thermal conductivity. While their thermal conductivity may be lower compared to that of pure metals and alloys, metal oxides offer advantages such as stable chemical properties, lower cost, and reliable performance. Consequently, metal oxides have been extensively researched as substitutes for pure metals. Babapoor et al. [54] used SiO_2_, Fe_2_O_3_, Al_2_O_3_, and ZnO nanoparticles to increase the thermal conductivity *k* of PCMs by 144%, 144%, 110%, and 110%, respectively. Through experiments, they concluded that Al_2_O_3_ and Fe_2_O_3_ nanoparticles played the most significant role in improving the thermophysical properties of PCM based on paraffin.

**Figure 4:**
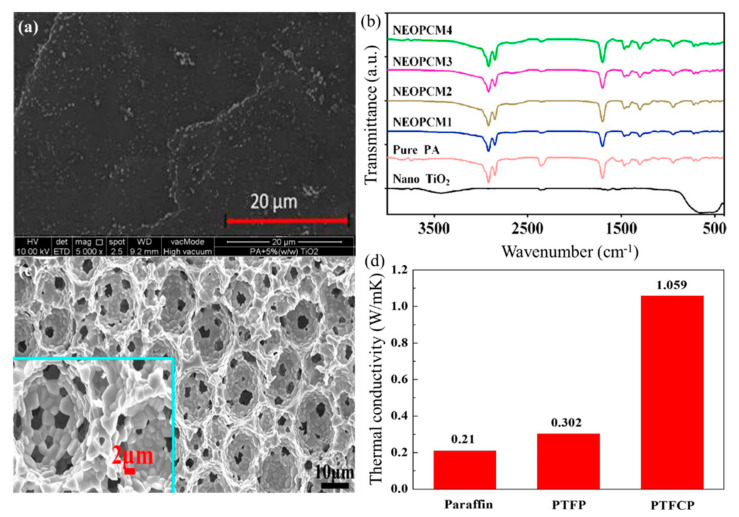
(**a**) FESEM image of nano TiO_2_-modified PCMs, (**b**) FT-IR spectra of nano TiO_2_-modified PCMs [55], (**c**) SEM image of nano TiO_2_ particle foam with carbon shell, and (**d**) thermal conductivities of the composite PCMs [56]. Reprinted/adapted with permission from Ref. [55]. Copyright 2016 Elsevier. Reprinted/adapted with permission from Ref. [56]. Copyright 2016 Elsevier.

Sharma et al. [55] studied the heat storage performance of synthetic TiO_2_ nanoparticle-modified palmitic acid (PA) composites. Figure 4a,b show the microstructure and FT-IR spectra of nano TiO_2_-modified PCMs, respectively. The thermal conductivity of the PCM composite was improved by 80% after being modified with nano-TiO_2_. The research also indicated that the modification effect had a linear relationship with the quantity of nano-TiO_2_ added. However, when nano-TiO_2_ was over-doped, it tended to agglomerate, leading to an inability to achieve the desired modification effect.

Li et al. [56] synthesized porous TiO_2_ foam through micro lotion technology, and burned it off after absorbing sucrose to solidify a layer of carbon shell on the surface of porous foam. Figure 4c shows a SEM image of nano TiO_2_ particle foam with a carbon shell. Compared with traditional TiO_2_, its thermal conductivity increased by 43.2%. The thermal conductivity of paraffin PCM was increased by 404% using carbon shell TiO_2_ nanoparticle foam, as shown in Figure 4d.

Sahan [57] synthesized nano-magnetite composites and dispersed them within paraffin PCM. Nano-Fe_3_O_4_ was prepared by low-cost sol–gel technology and mixed into paraffin at ratios of 10 wt% and 20 wt%. Fe_3_O_4_ nanoparticles with a diameter of 40–70 nm were prepared by sol–gel technology using ferric chloride hydrate ((FeCl_3_·6H_2_O, FeCl_2_·4H_2_O), dilute hydrochloric acid, and ammonia), and oleic acid was used to cover the surface to reduce particle aggregation. Iron oxide nanoparticles could be uniformly dispersed in the paraffin matrix. The *k* value increased by 48% and 60%, respectively, when 10 wt% and 20 wt% nano-magnetite were added. The research proved that incorporating nano-magnetite particles into PCM is an efficient and economical method to enhance thermal conductivity.

Jiang et al. [58] used nano Al_2_O_3_ to modify phase change microcapsules through lotion polymerization. The thermal conductivity of PCM microcapsules increased from 0.245 W/m·K to 0.38 W/m·K (an increase of 55%), and the *k* value of PCM microcapsules was greatly improved. Experimental data showed that the addition of nano Al_2_O_3_ was almost proportional to the enhancement of thermal conductivity.

#### 3.1.3. Carbon Nanomaterials

Carbon materials possess the greatest thermal conductivity, which is well over that of metals and metal oxides. The thermal conductivity of carbon nanotubes, graphene, and graphite is about 5-fold higher than that of silver. As the synthesis of carbon nanomaterials is becoming easier, research on using carbon nanomaterials to improve the thermal conductivity of PCMs is also increasing. Carbon nanomaterials commonly used for the modification of PCMs include carbon nanotubes [59], graphene [60], graphene oxide [61], carbon nanofibers [62,63], etc.

Ji et al. [64] proved that the thermal conductivity of PCM doped with 1.2 vol% of connected ultra-thin graphite foam (UGF) could be increased by 18 times, and the specific heat of melting or melting temperature did not change significantly. Graphite foam is composed of ultra-thin graphite; it has better thermal performance and thermal response and the thermal conductivity of this nanomaterial is greater than that of solid metal and carbon foam.

**Figure 5:**
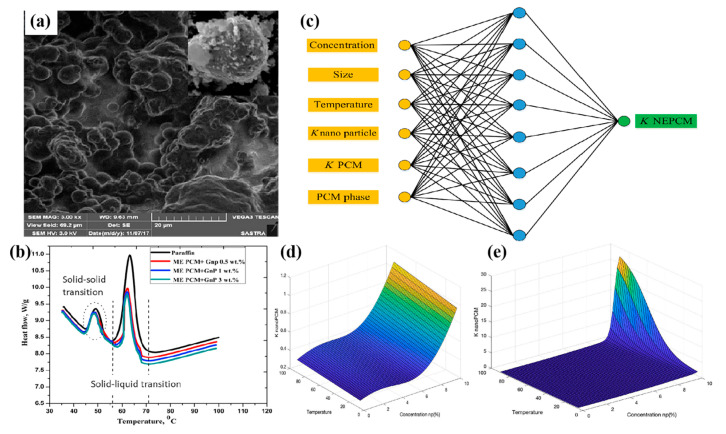
(**a**) SEM image of graphene-modified microencapsulated polyurethane–paraffin PCM (The scale represents 20 μm), (**b**) DSC curves of graphene-modified microencapsulated polyurethane–paraffin PCM [66], (**c**) design of the developed ANN model, (**d**) three-dimensional plot illustrating the thermal conductivity of NEPCM in the solid phase as a function of temperature and concentration, and (**e**) three-dimensional plot depicting the thermal conductivity of NEPCM in the liquid phase as a function of temperature and concentration [69]. Reprinted/adapted with permission from Ref. [66]. Copyright 2019 Elsevier. Reprinted/adapted with permission from Ref. [69]. Copyright 2023 Elsevier.

Shi et al. [65] increased the thermal conductivity of paraffin wax by 1000% using flake graphite nanoflakes (xGnP) and increased the thermal conductivity of paraffin wax by 100% using graphene. The thermal conductivity of paraffin PCM with a stable shape could be improved by adding xGnP and graphene. A small amount of xGnP and graphene could be used together as a low-cost enhancer to enhance heat dissipation. Praveen et al. [66] employed nano-sheet graphene with a thickness of 5 to 20 nm to improve the thermal conductivity of microencapsulated polyurethane–paraffin. Figure 5a,b show the microstructure and DSC curves of graphene-modified microencapsulated polyurethane–paraffin PCM. Adding 3 wt% nano-particles improved the thermal conductivity of the PCM by 97.4%. Oya et al. [67] dispersed exfoliated graphite into erythritol to form a composite PCM, by which the thermal conductivity of the PCM increased by 540%. Cui et al. [68] used carbon nanofibers (CNFs) to increase the thermal conductivity of soybean wax PCM by 44%. Through multiple experiments, it was found that the modification effect was approximately linear to the amount of CNF. By comparing the modification effects of multi-walled carbon nanotubes and CNFs, it was found that CNFs had certain advantages in enhancing the thermal conductivity of paraffin PCMs.

An Artificial Neural Network (ANN) and Response Surface Method (RSM) were presented to develop a model for predicting the thermal conductivity of carbon-based nano-enhanced phase change materials (NEPCMs) based on 482 experimental samples collected from various datasets. The ANN featured a multi-layered feed-forward structure and employed the Levenberg–Marquardt back-propagation algorithm, with one hidden layer containing eight neurons. The architecture of the ANN model is shown in Figure 5c. According to Figure 5d,e, it is noteworthy that at a 10% concentration and with a temperature range from 10 °C to 90 °C, the thermal conductivity changes of the nano PCM in the liquid phase were considerably higher than those in the solid phase [69].

In general, prediction methods for the thermal conductivity of nano-enhanced phase change materials (PCMs) based on artificial intelligence (AI) are a future development trend. AI predictions can more efficiently screen nanomaterials, optimize doping levels, and develop potential hybrid and novel nanomaterials, thereby significantly enhancing the thermal conductivity of phase change materials.

### 3.2. Latent Heat

#### 3.2.1. Influence Principles

The properties of nanomaterials are excellent; they have good thermal and chemical stability and their melting point is usually much higher than the phase transition point of PCMs. In addition, nanomaterials usually have high thermal conductivity; the specific heat capacity is also very low and the sensible heat storage capacity is almost negligible. Therefore, when nanomaterials are mixed into PCM systems, it can be considered that the nanomaterials themselves have no heat storage capacity, which often leads to the formation of nanoparticle–PCM composites with lower latent heat than pure PCMs [21].

However, in essence, the generation of the latent heat of PCMs is the heat that needs to be absorbed when they overcome intermolecular forces during melting. Therefore, theoretically speaking, when nanomaterials are doped into a PCM system, if there is enough interaction between them, the PCM needs to overcome its own intermolecular force and also the interaction between nanomaterials in the phase change process, thus showing a greater latent heat value [70]. However, so far, there is still controversy about the influence of nano-modification on the latent heat of PCMs. Scholars often produce different results by different processes even though they use the same kind of materials. The following is a list of the effects of different types of nanomaterials on the latent heat of PCMs.

#### 3.2.2. Carbon Nanomaterials

There are two types of intermolecular attraction: one is the short-distance force acting when the distance between the molecular centers is less than or equal to 3Å and the other is the long-distance force acting when the distance is larger. Generally, the short-range force is repulsive, while the long-range force or van der Waals force is attractive if there is no chemical interaction between molecules [71]. The chemical potential energy in the weak van der Waals attraction in the ionic bond and covalent bond is called the latent heat of the material. During the solid–liquid phase transition, the absorbed heat energy is employed to overcome the weak attraction between molecules, rather than the temperature rise. This energy destroys the solid bond, leaving energy related to the liquid intermolecular force [72].

Therefore, if the interaction between nanomaterials and PCMs is greater than that between PCMs, the latent heat will increase. According to this theory, the latent heat of PCMs is easy to change when the concentration of nanoparticles is high and the specific surface area is large [73].

Recently, many scholars have found that adding carbon nanotubes into PCMs causes changes in latent heat. Shaikh et al. [74] studied the effects of SWCNTs (single-walled carbon nanotubes), MWCNTs (multi-walled carbon nanotubes), and CNFs on the phase transition behavior of paraffin and found that the maximum latent heat of paraffin–SWCNT composites increased by 13%. Based on the Lennard-Jones potential, the paper discussed the change of latent heat using the approximate value of intermolecular gravity and obtained the relationship between the change of latent heat and the diameter of carbon nanotubes. The study found that the latent heat of PCMs was roughly inversely proportional to the diameter of carbon nanotubes, which may be related to the stronger interaction between carbon nanotubes and PCMs when the diameter of carbon nanotubes is lower.

Karaipekli et al. [75] summarized the following (Table 1) through experiments. It can be seen from Table 1 that the increase in carbon nanotube content aggravates the loss of latent heat.

#### 3.2.3. Nano-Metal and Metal Oxide

There are different results on the influence of metal and metal oxide nanomaterials on latent heat. Elbahjaoui and Hamid El Qarnia [76] added 2–8 vol% copper nanoparticles to n-octadecane, and the results showed that the presence of nanoparticles increased the latent heat. They made an analysis of the latent heat release of PCMs, but did not explain the mechanism of nanomaterials increasing the latent heat of n-octadecane. Rufuss et al. [77] added 3 wt% TiO_2_, CuO, and GO into a paraffin material, respectively. The research showed that GO could reduce the latent heat, but CuO and TiO_2_ nanoparticles increased the latent heat. They attributed the change of latent heat to the surface charge state of nanoparticles, the delamination in the liquid–solid phase, and the mechanism of phonon motion.

However, Praveen et al. [78] added 0.5~3% weight of CuO nanoparticles to neopentyl glycol (NPG), and the enthalpy decreased by 3.1 to 9.6%. The experiment found that the increase in the weight ratio of CuO nanoparticles led to a decrease in the latent heat value of the composite sample. Ho and Gao [79] also found that compared to pure paraffin, the enthalpy of paraffin nanoparticle emulsions decreased significantly with increasing mass fractions of nanoparticles when modified with alumina nanomaterials. For paraffin containing 5 wt% and 10 wt% nanoparticles, the melting latent heat decreased by more than 7% and nearly 13%, respectively. Zeng et al. [80] also found that the latent heat of composite PCMs decreased with the increase in the mass fraction of Cu nanowires when using Cu nanowires to modify tetradecanol, and they believed that the loss was acceptable. Salyan and Suresh [81] also observed that the latent heat of PCMs decreased linearly with an increase in the mass fraction of nano-copper oxide when they modified D-mannitol with nano-copper oxide.

**Figure 6:**
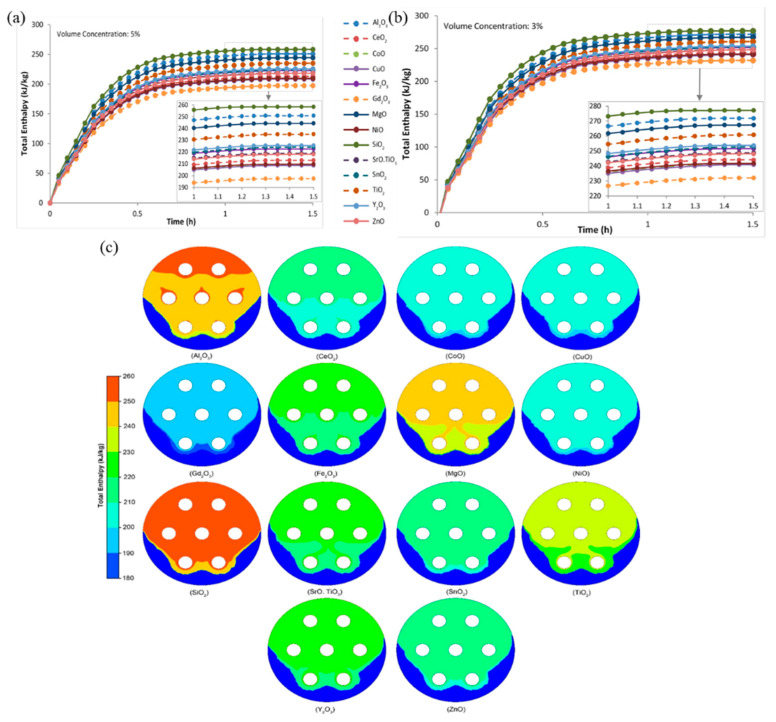
Total enthalpy of nano-PCM with 5 vol% (**a**) and 3 vol% (**b**) of different types of metallic oxides and (**c**) enthalpy contours of nano-PCM samples with 5 vol% metallic oxides after charging for 25 min [82]. Reprinted/adapted with permission from Ref. [82]. Copyright 2019 Elsevier.

Zakir Khan et al. [82] conducted a detailed evaluation of heat transfer in a composite PCM latent heat storage system based on nano-metal oxides. Figure 6a,b display the total enthalpy of nano-PCMs with different types of metallic oxides, while Figure 6c illustrates the enthalpy contours of nano-PCM samples with a 5% volume percentage of metallic oxides after charging for 25 min. It was observed that nano-PCM samples based on Al_2_O_3_, MgO, and TiO_2_ consistently exhibited higher total enthalpy, ranging from 297.8 to 291.2 kJ/kg. However, when compared with the base paraffin, their latent heat decreased, with the percentage of total enthalpy decreasing from 3.12% to 5.27%. Conversely, the total enthalpy of the nano-PCM composites with 3 vol% and 5 vol% Gd_2_O_3_ decreased by 24.53% and 35.78%, respectively. The thermophysical properties of nanoparticles, such as density, thermal capacity, and thermal conductivity, had a significant impact on the total enthalpy of the PCM composite. Metal oxide nanoparticles with lower density and greater thermal conductivity and heat capacity were considered optimal alternatives for improving the enthalpy.

Şahan et al. [57] utilized nano-magnetite to modify paraffin PCM, and the results showed that the latent heat of composite PCMs with 10% and 20% nano-magnetite increased by 3.3% and 8.8%, respectively, compared with pure paraffin. The slight increase or unchanged latent heat storage capacity of nano-composite PCMs could be attributed to the large surface-to-volume ratio of the nanomaterials. This effect can be explained using an approximation of the intermolecular attraction from the Lennard-Jones potential between nanoparticles and paraffin.

### 3.3. Specific Heat Capacity

The main factors influencing the specific heat capacity of different types of PCMs in various states differ. For instance, in crystalline PCMs such as hydrated salts, the specific heat in the solid state is related to the interfacial thermal resistance within the crystals. This resistance mainly occurs between the grains, meaning that the number of grains in PCMs partially reflects their specific heat capacity. When nanomaterials are introduced, they can produce more grains during crystallization, thereby increasing the specific heat capacity [83,84]. However, for some amorphous PCMs, the introduction of nanomaterials is equivalent to incorporating materials with lower specific heat. In this case, the specific heat of the composite should be calculated based on the density and specific heat of the materials [85]. In general, the specific heat of composite PCMs prepared by modifying organic PCMs with nanomaterials is lower than that of pure PCMs.

**Figure 7:**
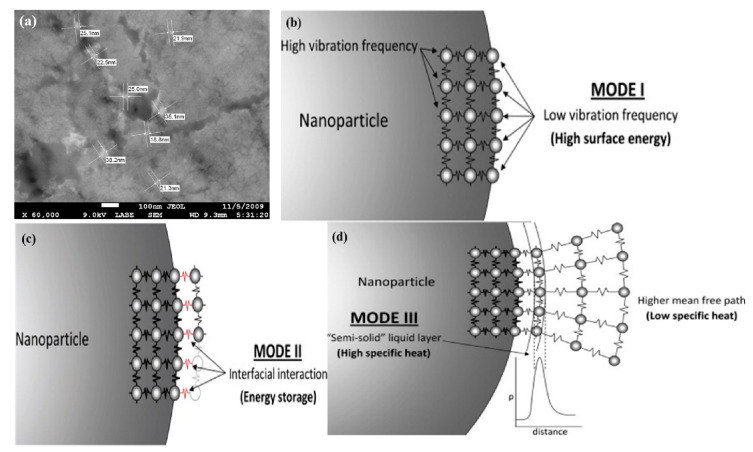
(**a**) SEM image of SiO_2_ nanofluid before melting and (**b**–**d**) three types of thermal transfer mechanisms [86]. Reprinted/adapted with permission from Ref. [86]. Copyright 2011 Elsevier.

Shin and Banerjee [86] studied and discussed the specific heat capacity of a chloride salt eutectic and its nanofluid obtained by adding 1 wt% SiO_2_ nanoparticles. The SEM image of the SiO_2_ nanofluid before melting is shown in Figure 7a. It was found that the specific heat of the composite phase change material increased by 14.5% compared with the pure chloride salt eutectic, which was significantly higher than the measurement uncertainty of 2–4%. Based on this phenomenon, they proposed three independent heat transfer mechanisms to explain the increase in specific heat capacity, as shown in Figure 7b–d.

(1) The specific heat capacity was increased because of the higher specific surface energy (compared to ordinary materials) of nanoparticles. (2) Due to the high specific surface area of nanoparticles, the interface interaction (such as interface thermal resistance) was enhanced. (3) There may have been semi-solid layers adhered to nanoparticles, which may have enhanced the specific heat capacity (compared to the higher intermolecular spacing in the control liquid material) due to their small intermolecular spacing, similar to the lattice structure on the surface of nanoparticles.

Chieruzzi and Cerritelli [87] studied the effects of three different nanomaterials on the thermal properties of molten salt PCMs. They concluded that the most likely mechanism leading to the increase in specific heat was the semi-solid layer formed by PCMs on the surface of nanoparticles. The specific heat of the nanofluids increased because the semi-solid layer exhibited greater thermal properties than other liquids. Dudda et al. [88] found that the specific heat of a binary nitrate eutectic increased with the incorporation of nanoparticles. They observed a reticular nano layer within the composite doped with 10 nm nanoparticles, which showed the most significant improvement in specific heat.

Rufuss et al. [77] added 3 wt% TiO_2_ and CuO to paraffin and found that the specific heat capacity decreased. They believed that the low specific heat capacity of the nanomaterials used led to the reduction of the specific heat of the nano-composite PCM. He [89] suspended TiO_2_ nanoparticles in a saturated BaCl_2_ aqueous solution, developing a new type of nanofluid PCM. During the experiment, they observed a decrease in specific heat resulting from the inclusion of nanoparticles. They identified two primary reasons for this phenomenon. Firstly, the specific heat of the nanoparticles influenced that of the composites. Secondly, the significant contribution of surface free energy to the system capacity, attributed to the large specific surface area of nanoparticles, also affected the specific heat of the composites. Tao and Liu et al. [90] demonstrated a contradictory result in the effect of carbon nanomaterials on the thermal storage performance of carbonate. Despite causing a reduction in latent heat, the nanomaterial additive improved specific heat and enhanced sensible heat storage capacity. This phenomenon was attributed to the high specific heat of the multi-walled carbon nanotubes used.

**Figure 8:**
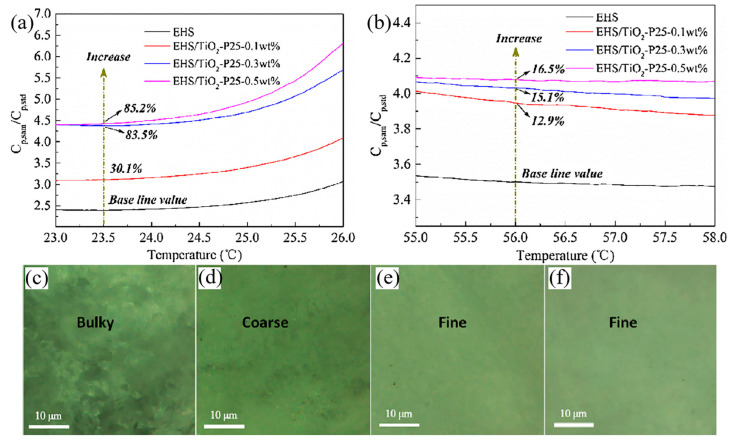
Relative specific heat of EHS and EHS/TiO_2_-P25 compared to sapphire (**a**) in solid state and (**b**) liquid state [91] and optical micrographs of (**c**) EHS, (**d**) EHS/TiO_2_-P25 (0.1 wt%), (**e**) EHS/TiO_2_-P25 (0.3 wt%), and (**f**) EHS/TiO_2_-P25 (0.5 wt%) in solid state [91]. Reprinted/adapted with permission from Ref. [91]. Copyright 2017 Elsevier.

Liu et al. [91] studied the effect of nano titanium oxide on the specific heat capacity of hydrated salt PCMs. Figure 8a,b show the relative specific heat capacity of EHS and EHS/TiO_2_-P25 nanofluids in the liquid state and solid state, respectively. It can be seen from Figure 8 that the specific heat capacity of EHS was significantly improved after adding TiO_2_-P25 nanoparticles to EHS. Compared with pure EHS, the specific heat capacity of 0.3 wt% TiO_2_-P25 nanoparticles in the solid state at 23.5 °C increased by 83.5% and reached 3.368 J/g·K. In order to explain the mechanism of specific heat capacity increase, Liu et al. [91] analyzed the optical microstructure of EHS and EHS/TiO_2_-P25 nanofluids containing 0.1 wt%, 0.3 wt%, and 0.5 wt% of TiO_2_-P25 nanoparticles. It can be seen from Figure 8c–f that with the increase in the TiO_2_-P25 nanoparticle content, the microstructure of EHS/TiO_2_-P25 nanofluids gradually went from rough to smooth, which was a process of grain refinement. The specific heat of solid crystalline materials depends on the interface thermal resistance. Moreover, greater interface thermal resistance increases the specific heat. Because the interface thermal resistance is generated by the interface between grains, it is not difficult to understand that for crystalline materials with a smaller grain size, the interface thermal resistance will be significantly improved due to the increase in interfaces.

**Figure 9:**
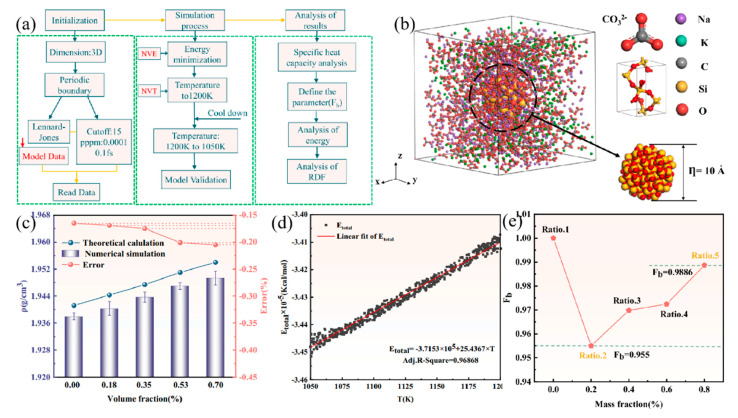
(**a**) The research process diagram, (**b**) structural arrangement of molten salt-based nanocomposite phase change materials, (**c**) calculated and simulated density values of NPCMs, (**d**) changes in the total energy of the NPCM system with temperature, and (**e**) evaluation of interaction effect indicators on the SHC of NPCMs [92]. Reprinted/adapted with permission from Ref. [92]. Copyright 2023 Elsevier.

The impact of SiO_2_ nanoparticles on the specific heat capacity (SHC) of molten salt was explored using the molecular dynamics (MDs) method, and the intrinsic mechanism behind the change in SHC was elucidated from a microscopic perspective. The research flow chart is illustrated in Figure 9a. A SiO_2_ supercell was created based on the original cell, and the desired spherical nanoparticle atomic cluster was extracted from the supercell. Na_2_CO_3_ and K_2_CO_3_ molecules were randomly distributed in the box at a molar ratio of 58:42, as depicted in Figure 9b. The densities of molten carbonate salts with varying nanoparticle concentrations were computed to verify the reliability of the simulated system, shown in Figure 9c,d. A parameter, Fb, was defined in the study to reflect the effect of the interaction between the nanoparticles and PCM on SHC. The reduction in the Fb value with increasing nanoparticle loading indicated an enhanced interaction, with the most significant interaction observed at a nanoparticle loading of 0.2 wt% [92]. The study offered a crucial theoretical approach to understanding the mechanism by which nanoparticles influence the specific heat capacity of phase change materials.

## 4. Medical Applications of Nano-Modified PCMs

Nano-PCMs utilized in biological medical applications often require a high latent heat of fusion and low melting temperatures, alongside low toxicity. Materials like stearic acid (SA), palmitic acid (PA), myristic acid (MA), capric acid (CA), and lauric acid (LA) are emerging as promising, abundant, and cost-effective candidates for various medical applications, given their favorable properties and versatility [26]. These applications include tissue engineering, drug delivery [93], healthcare [94], and medical dressing [95], among other uses.

**Figure 10:**
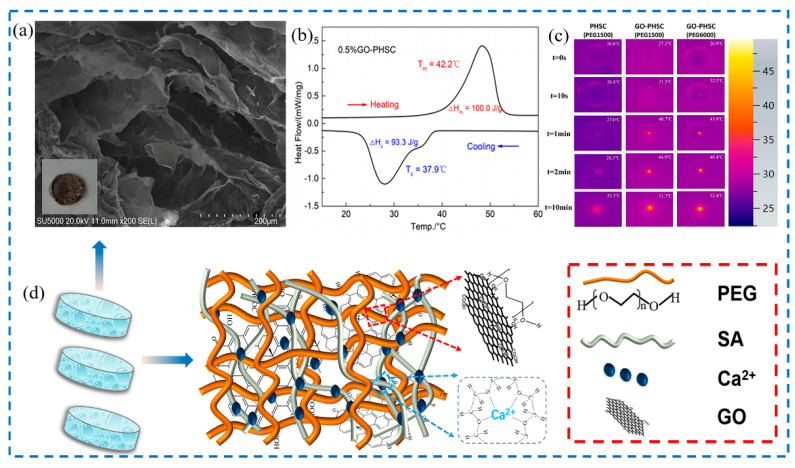
(**a**) Microstructure, (**b**) thermal properties, and (**c**) temperature control performance of PHSC with 0.5 wt% GO content and (**d**) synthesis mechanism diagram of GO-PHSC [96]. Reprinted/adapted with permission from Ref. [96]. Copyright 2024 Elsevier.

Zhang et al. [96] conducted research on the application of PCMs in bone tissue engineering. They innovated a novel form-stable PCM embedded in a hydrogel scaffold for precise temperature control during photothermal therapy. Polyethylene glycol (PEG) hydrogel scaffolds (PHSCs) were synthesized via semi-crosslinking with calcium-ion-chelated sodium alginate (SA) as the framework, utilizing an ion coordination crosslinking method, and the synthesis mechanism is shown in Figure 10d. The integration of 0.5 wt% graphene oxide (GO) improved the microstructure (Figure 10a) and enhanced the thermal properties of the nano PCMs, exhibiting a high thermal energy storage capacity of 100.0 J/g, with a satisfactory latent heat recovery of 93.3%, and an ideal phase change temperature of 42.2 °C (Figure 10b). Additionally, the addition of PEG1500 significantly buffered the heating rate around 42 °C, which is conducive to bone formation, as shown in Figure 10c.

Nano-PCMs with melting temperatures close to the human body temperature are ideal for delivering thermo-sensitive medicines. Certain drugs are only released at specific temperatures, and PCMs can be used to achieve drug release by generating particular temperatures within the body. Additionally, PCMs can control the rate and quantity of drug release through slight temperature variations. This enables the drug to undergo therapeutic treatment on affected tissues over a specific period at a predetermined rate [8]. A drug delivery system based on PCMs holds significant promise for enhancing the effectiveness of chemotherapy, reducing side effects, and improving biosafety. PCMs act as “gatekeepers” in the development of such drug delivery systems, responding to temperature changes. When combined with photo-thermal conversion agents such as metal nanoparticles and carbon nanoparticles, PCMs can lead to the creation of a nanoscale release system. Gold nanoparticles, in particular, have garnered significant attention due to their high energy conversion efficiency and biocompatibility [97].

In a previous study, precious metals with a hollow nanostructure, such as gold nanocages (AuNCs), emerged as promising nanocarriers. Researchers successfully addressed the challenge of packaging both hydrophilic and hydrophobic drugs synergistically by incorporating organic PCMs containing anticancer drugs into the hollow interior of AuNCs [98,99]. For example, Xia et al. [100,101] successfully incorporated various therapeutic agents, including anticancer drugs such as DOX and H_2_SeO_3_, into organic PCMs. Following efficient loading, these PCMs were further encapsulated within the interior of gold nanocages (AuNCs). Serving as the shell of the nanodrug carrier, AuNCs also functioned as photothermal transducers, capable of absorbing near-infrared (NIR) light and converting it into heat. The therapeutic agents loaded onto AuNCs were typically pre-released, eliminating them before accumulating at the tumor site. The introduction of PCMs with melting points of around 40 °C allowed these potent therapeutic agents to maintain their excellent therapeutic activity prior to reaching the targeted lesion. Additionally, researchers modified AuNCs to enhance the precision of targeted therapy. The surface modification of nanoscale drug carriers with targeting ligands proved to be an effective strategy for increasing the accumulation of nanocarriers in the specified target region [102].

**Figure 11:**
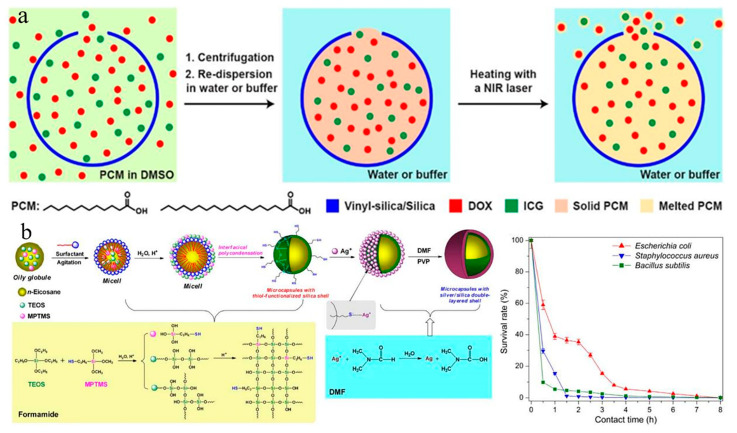
(**a**) Schematic representation of the encapsulation of PCM within nanocapsules featuring an open pore for the temperature-regulated release of drugs [105]. (**b**) Illustration of the synthetic strategy for multifunctional microcapsules with an n-eicosane core and a silver/silica double-layered shell. The survival rates of various bacteria are presented as a function of contact time for the silver/silica double-layered microcapsules obtained with an 8-h reaction time [107]. Reprinted/adapted with permission from Ref. [105]. Copyright 2019 John Wiley and Sons. Reprinted/adapted with permission from Ref. [107]. Copyright 2016 Elsevier.

As a biocompatible material, SiO_2_ nanoparticles possess a structure that allows easy modification by various functional groups. Moreover, these nanoparticles can undergo self-removal from the human body. Consequently, nano-SiO_2_ is considered an advantageous modified material for enhancing the biocompatibility of PCMs [103,104]. For instance, Qiu et al. [105] introduced a nanocapsule PCM based on nano-SiO_2_, serving as a temperature-controlled drug release platform. The fabrication process of the nanocapsule PCM is depicted in Figure 11a. The nanocapsule, featuring well-defined holes, was crafted through site-selected deposition, and its nano-SiO_2_ shell encapsulated a PCM derived from fatty acid. This innovative approach resulted in the heightened stability and improved biocompatibility of the drug carrier system.

Silver ions have the ability to establish robust binding bonds with certain substances utilized by microorganisms in their respiration, including molecules containing oxygen, sulfur, and nitrogen. This interaction prevents microorganisms from utilizing these substances, leading to their asphyxiation and eventual demise [106]. The utilization of silver ions for modifying phase change materials (PCMs) imparts antibacterial properties to the PCMs, thereby extending the clinical applications of these materials.

Zhang et al. [107] innovatively synthesized a microcapsule featuring an n-eicosane PCM core and a silver/silica double-layered shell. The manufacturing process involved the interfacial condensation of silica precursors with thiol groups in a non-aqueous emulsion templating system. Subsequently, silver ions were reduced and deposited onto the surface of the silica shell of the microcapsules. This microcapsule demonstrated notable antibacterial activity, particularly against Staphylococcus aureus and Bacillus subtilis. Impressively, the sterilization rate surpassed 95% after a 4-h contact period with these bacteria (as illustrated in Figure 11b). The integration of PCM with silver nanoparticles in a medical thermostatic bandage has proven effective in mitigating the risk of wound inflammation and infection caused by high temperatures in summer. This application plays a pivotal role in positively influencing the wound-healing process for patients [108].

At present, there are no commercially available phase change materials (PCMs) for cancer treatment, although some formulations are in the early stages of clinical trials, showing promising development trajectories. PCMs have extensive applications in the textile industry, where they serve as encapsulating materials or fibers that absorb and release heat based on body and environmental temperatures, preventing sweating and freezing. Textiles endowed with thermal storage and temperature control characteristics can maintain skin temperatures within a comfortable range, making them suitable for use as bandages in burn and cold/heat treatments. The incorporation of PCM with silver nanoparticles in medical thermostatic bandages has demonstrated efficacy in reducing the likelihood of wound inflammation and infection during high temperatures in summer, thereby positively contributing to the wound-healing process of patients [108]. Additionally, fabrics treated with polyethylene glycol (PEG) are utilized in hygiene products such as diapers and incontinence products. These fabrics offer a combination of fluid delivery and antimicrobial properties, making them highly desirable for maintaining cleanliness and comfort [109].

Chen et al. [9] engineered a multifunctional mask incorporating CNT and PEG. The porous framework of a CNT-based aerogel functioned as a particulate catcher, while the CNT-based aerogel/PEG composite served as a thermal regulator. This mask facilitated the delivery of air at temperatures ranging from 40 to 43.5 °C into the nasal cavity for 30 min. Notably, in the thermotherapeutic group, there was a significant decrease observed in nasal secretion eosinophils.

**Figure 12:**
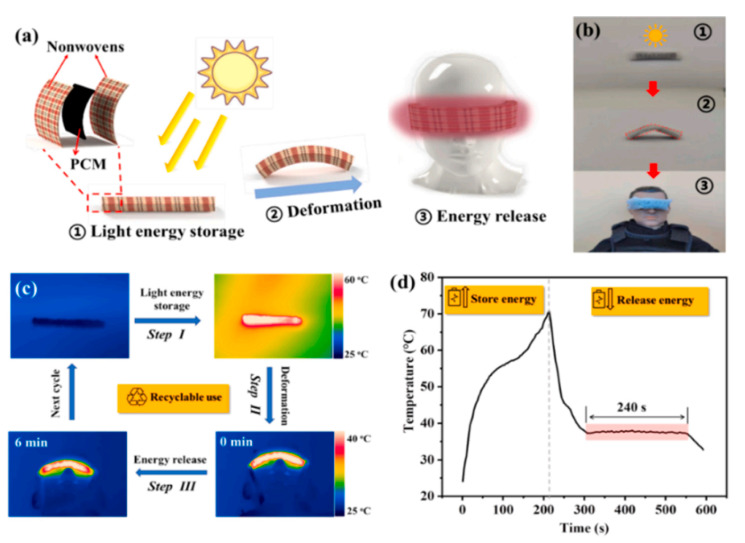
(**a**) Schematic diagrams delineate the composition and operational procedures of the thermal eye patch. (**b**) Photographic representations demonstrate the excellent flexibility of the thermal eye patch and its comfortable application on human eyes. (**c**) Infrared thermal images depict the temperature variations during different stages of the thermal eye patch. (**d**) The temperature evolution curve of the thermal eye patch is illustrated under both NIR light irradiation and when turned off [110]. Reprinted/adapted with permission from Ref. [110]. Copyright 2021 Elsevier.

In another work [110], advanced PCM composites were developed, exhibiting excellent encapsulation properties, superior solar-to-thermal conversion efficiency, and a shape memory function. This was achieved by incorporating MXene-coated melamine foam (MF@MXene) into PEG. The resulting MF@MXene/PEG composites demonstrated impressive attributes such as a high dimension retention ratio (90%) and significant phase change enthalpy (194.1 J/g). Enhanced light absorbance was achieved in the PCM composites through the addition of a MXene scaffold, resulting in a remarkable solar energy conversion efficiency of 92.7%. These light-responsive PCM composites showcased a light-actuated shape memory function, achieving a nearly 100% shape recovery ratio. Moreover, as depicted in Figure 12, the PCM composites were utilized as flexible heat eye patches, forming a snug fit with human eyes and consistently providing thermal comfort within a narrow temperature range.

**Figure 13:**
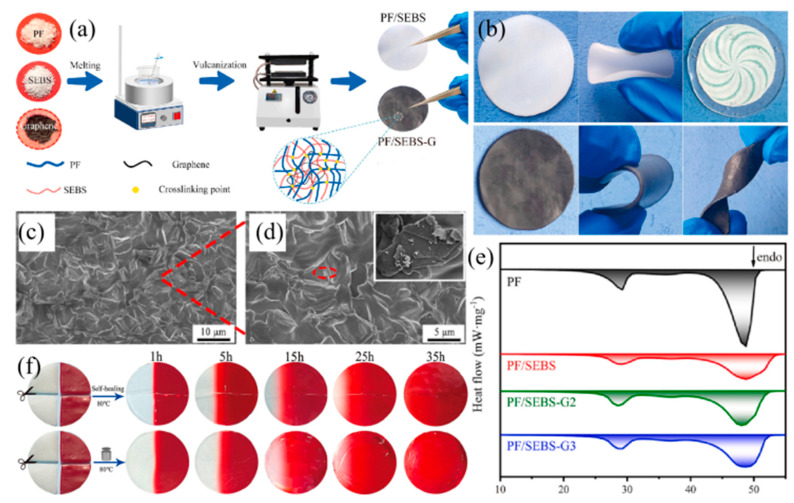
(**a**) Schematic diagram showing the fabrication process of PF/SEBS and PF/SEBS-G, (**b**) photographs demonstrating the bending and folding of PF/SEBS and PF/SEBS-G3, (**c**,**d**) images of the fracture surfaces of PF/SEBS-G3, (**e**) DSC curves depicting the melting process of various samples, and (**f**) photographs illustrating the healing process of stained PF/SEBS [113]. Reprinted/adapted with permission from Ref. [113]. Copyright 2024 Elsevier.

Medical dressings are vital for wound care, as they play a key role in creating an optimal environment for healing. Maintaining a consistently warm temperature at wound sites is crucial, as this can enhance blood flow and alleviate pain. Dressings with temperature-regulating properties can help achieve this. Additionally, preventing wound infections is essential for successful healing. This can be achieved through the use of dressings with hemostatic properties to control bleeding, antibacterial coatings to inhibit microbial growth, and controlled wet dressings to maintain an appropriate level of moisture for healing [111]. In a study by Zhang et al. [112], a thermosensitive drug-releasing fiber hydrogel dressing was developed to expedite the healing of skin wounds. Nanofibrous hydrogels were impregnated with fatty acids/aspirin (ASP)-based polydopamine (PDA), demonstrating a rapid release of ASP at a temperature of 40 °C. Results from animal experiments indicated the favorable application of these temperature-responsive nanofibrous hydrogels in wound care. To provide thermal compression dressings with multifunctionality and applicability in various scenarios, flexible films were developed using paraffin (PF) as a thermal energy storage medium, a styrene–ethylene/butylene–styrene (SEBS) triblock copolymer as the matrix, and graphene as a photo-thermal conversion agent (Figure 13a). The presence of PF/SEBS-like structures and lamellar graphene, shown in Figure 13c,d, confirmed the successful integration of graphene into PF/SEBS. These films exhibited remarkable flexibility and self-healing properties, making them suitable for different body parts and reusable in practical applications, thus significantly reducing the cost of treating chronic joint conditions. Both PF/SEBS and PF/SEBS-G3 could be freely bent and folded, with PF/SEBS becoming transparent when heated, as shown in Figure 13b. This flexibility ensured that the films conformed well to the skin during heat application. According to the DSC curves in Figure 13e, the PF/SEBS-G3 film containing 77 wt% PF completely melted at 52.22 °C, with a melting enthalpy of 157.26 J/g. Experimental results in Figure 13f show that the red dye spread throughout the composite after 35 h, indicating sufficient molecular movement during the healing process. For PF/SEBS healed under pressure, fracture marks began to fade within 1 h and disappeared completely by 5 h, whereas PF/SEBS relying on self-healing still showed visible fracture marks after 35 h. Considering both healing speed and effectiveness, the pressure-assisted healing method proved to be more efficient. In summary, the PF/SEBS-G3 thermal patch demonstrates significant potential for thermal therapy applications [113].

Cold chain logistics are essential for preserving the integrity of temperature-sensitive products like fresh produce, medications, and vaccines, thereby playing a crucial role in maintaining human health and well-being [114]. Ensuring temperature stability throughout the cold chain logistics process is complex, as products traverse through a labyrinth of interconnected systems within the supply chain, each with its own unique set of conditions and requirements [115].

**Figure 14:**
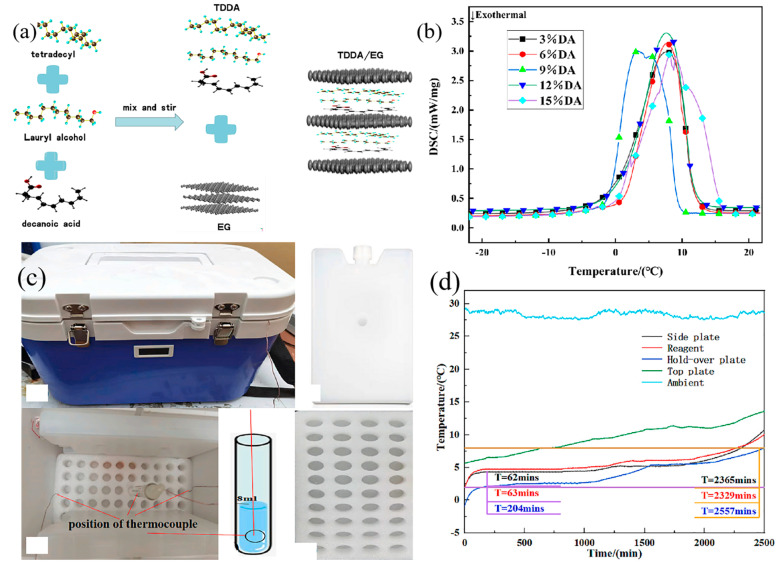
(**a**) Diagram illustrating the preparation mechanism, (**b**) diagram depicting the endothermic curve of DSC, (**c**) schematic representation of a cold storage box, and (**d**) graph illustrating the temperature variation in the cold storage box [116]. Reprinted/adapted with permission from Ref. [116]. Copyright 2023 Elsevier.

A cold storage phase change material named tetradecane–dodecanol–decanoic acid (TDDA) was developed specifically for maintaining the temperature range required for medical and food logistics, which is between 0 and 8 °C. This material was composed of tetradecane, capric acid, and dodecanol, as illustrated in the preparation mechanism shown in Figure 14a. In Figure 14b, it is demonstrated that PCCSM containing 12% DA possessed a higher latent heat of phase transition and achieved the desired transition temperature range of 0 to 5 °C without encountering phase separation issues. The schematic diagram of the cold storage box is presented in Figure 14c. By conducting experiments with the cold storage box, the temperature fluctuations of vaccines were analyzed. The average duration of cold preservation in the cold storage box employing TDDA/EG material was 37.8 h, which was 50.4 times longer than that of the cold box lacking TDDA, as depicted in Figure 14d [116]. In conclusion, TDDA/EG phase change cold storage materials exhibit promising prospects for the storage of temperature-sensitive products in the vaccine cold chain.

In Table 2, we summarize the applications of PCMs in the medical field, including types of PCMs, application scenarios, and specific descriptions.

## 5. Current Challenges and Future Trends

Based on the above analysis, the current challenges and future trends are marked as follows. At present, the research on nano reinforcement mainly focuses on the improvement of heat transfer performance, and shows excellent modification effects. However, the influence mechanism of nanomaterials on latent heat and specific heat has not been fully determined. It is worth noting that the effect of nanomaterials on the latent heat of PCMs often shows opposite conclusions, but most of the studies show that the latent heat of PCMs is reduced with increasing nanomaterial content. For PCMs used in thermal energy storage systems, the latent heat mainly determines the thermal energy storage capacity, but, in order to obtain higher efficiency of heat charging and discharging, it often causes the partial loss of thermal energy storage capacity with the addition of nanomaterials. When nanomaterials are used to modify PCMs, both high thermal conductivity and high heat storage cannot be achieved at the same time. Considering the high price of nanomaterials, the performance improvement of PCMs is not always positive when used. Therefore, we need to focus on the pros and cons. In addition, in some research reports, nanomaterials have also been used to improve the latent heat of PCMs, but the current conclusions cannot provide a completely effective theoretical method to improve the latent heat of PCMs or a perfect mechanism explanation. Therefore, the future research focus needs to pay more attention to the summary of the work on nanomaterials to enhance the latent heat, reveal its reinforcement mechanism, and improve the relevant theory to maximize the use of nanomaterials to improve the thermophysical properties of PCMs. In addition, the effectiveness of enhancing the specific heat capacity of PCMs using nanomaterials still has room for improvement. Future research could focus on designing and optimizing nanostructures to maximize their interface area with PCMs, thereby improving specific heat capacity. This may involve selecting and combining different types of nanomaterials such as nanoparticles, nanowires, and nanosheets.

Through nanomaterial enhancement modification, the biocompatibility of PCMs can be improved, reducing interactions with the biological system. In the design of a controllable drug delivery system, the introduction of nanomaterials enables more precise control over the drug release rate. By regulating the thermal properties of nanocomposite PCMs, consideration is given to adjusting the temperature sensitivity, enhancing the ability of PCMs to control temperature and release in complex physiological environments. Exploring the modulation of the response properties of PCMs to external stimuli such as temperature and light using nanomaterials can optimize response speed, sensitivity, and controllability to meet the requirements of various biomedical applications. Utilizing nanomaterial enhancement modification to achieve the multifunctional enhancement of PCMs, including the integration of photothermal/magnetic hyperthermia treatment, bioimaging, and other functions, should be considered. The incorporation of various nanomaterials can be used to achieve a broader range of biomedical applications.

## 6. Conclusions

The concept of dispersing nanoparticles to enhance the thermal properties of PCMs has gradually matured. However, the impacts of nanoparticles on the thermal behavior of PCMs need to be further discussed. In addition, although dispersed nanoparticles possess several front effects on the thermal performance of PCMs, their side effects still need to be taken seriously. Moreover, the application of nano-modified PCMs in the medical field is also under investigation. Through this summary and analysis, the following conclusions are obtained:

(1) Adding nanomaterials to PCMs can generally improve the thermal conductivity of PCMs. The heat conductivity of nano-modified PCMs increased significantly with the increase in nanoparticle concentration in PCMs. Among many nanomaterials, CNT, CNF, graphene, and other carbon nanomaterials have excellent physical properties, and the modification effect is far better than that of other nanomaterials. The modification effect of its heat transfer performance is related to its size, dosage, and surface properties.

(2) The enhancement effect of metal and metal oxide nanoparticles on the thermal conductivity of PCM changes with changes in shape, size, and concentration. Compared with pure metals, metal oxides are cheaper and more stable and are easier to popularize.

(3) The increase in thermal conductivity greatly reduces the crystallization and melting time of composite PCMs. Different nanoparticles show different strengthening effects on the crystallization of PCMs. In most cases, the crystallization and melting of nano-enhanced PCMs depend on the mass/volume concentration of dispersed nanoparticles. However, when the concentration of nanoparticles reaches a certain level, it not only agglomerates but also has side effects on other performances of PCMs. Therefore, nanomaterials cannot be introduced into PCMs without a dose limit.

(4) Generally speaking, the latent thermal capability of PCMs decreases with the increase in nanoparticle concentration, though it may increase in special cases. The diverse behaviors of nanoparticles influence the reduction of latent thermal capacity in different ways. There should be an optimal concentration level of nanoparticles, in which the enhancement of thermal conductivity is the largest and the reduction of latent heat capacity is the smallest.

(5) The influence of nano-modified PCMs on the specific heat capacity mainly depends on three aspects. The first is the relative size of the specific heat capacity of PCMs and nanomaterials. The second is whether the size of the nanomaterials can form plate-like and network nanostructures. The third is whether the specific surface area of the nanomaterials can increase enough free energy.

(6) Nano-PCMs employed in biological medical applications showcase a notable latent heat of fusion and low melting temperatures, all while maintaining good biocompatibility. The versatility and advantageous properties of PCMs, achieved through nanoenhancement and nanofunctionalization, make them suitable for a diverse range of medical applications, including drug delivery, healthcare, medical dressing, and so on.

## Figures and Tables

**Table 1:** Change of latent heat of different types of PCMs [75].

Materials	Heating		Cooling	
	Melting Temperature (°C)	Melting Latent Heat (J/g)	Crystallization Temperature (°C)	Crystallization Latent Heat (J/g)
C20	36.18	275.91	32.92	268.13
ExP/C20 (60%)	36.12	161.18	34.32	155.26
ExP/C20 (60%)/Carbon nanotubes (0.3 wt%)	36.24	160.38	34.32	155.26
ExP/C20 (60%)/Carbon nanotubes (0.5 wt%)	36.34	159.02	35.55	145.92
ExP/C20 (60%)/Carbon nanotubes (1 wt%)	36.47	157.43	35.53	141.15

**Table 2:** Medical applications of PCMs.

PCM	Application	Description	References
Polyethylene glycol (PEG)	Tissue Engineering	PCM embedded in hydrogel scaffold for temperature control during photothermal therapy.	[96]
Lauric acid (LA)	Drug Delivery	Utilization of PCMs for precise temperature-controlled drug release, enhancing therapeutic efficacy.	[98]
N-eicosane PCM	Antibacterial Dressings	Incorporation of silver-modified PCMs in medical bandages for wound care, reducing infection risks.	[107]
Polyethylene glycol (PEG)	Hygiene Products	PCM-treated fabrics in hygiene products like diapers for moisture control and antimicrobial benefits.	[109]
Polyethylene glycol (PEG)	Thermotherapy Masks	CNT-based aerogel/PEG composites in masks for thermotherapy, offering relief from nasal conditions.	[9]
Polyethylene glycol (PEG)	Thermal Eye Patches	Flexible PCM composites for thermal eye patches, ensuring comfort and temperature control.	[110]
Fatty acids (FAs)	Wound Healing Dressings	Temperature-responsive PCM hydrogel dressings for promoting wound healing.	[112]
Paraffin	Thermal Therapy Films	PF/SEBS-G3 films with graphene for thermal therapy, featuring flexibility and self-healing properties.	[113]
Tetradecane–dodecanol–decanoic acid (TDDA)	Cold Chain Logistics	TDDA/EG PCM for maintaining temperature stability in cold storage boxes during the transportation of vaccines and medical products.	[116]

## Data Availability

The original contributions presented in the study are included in the article, further inquiries can be directed to the corresponding author.
